# Transcriptional regulation of thylakoid galactolipid biosynthesis coordinated with chlorophyll biosynthesis during the development of chloroplasts in *Arabidopsis*

**DOI:** 10.3389/fpls.2014.00272

**Published:** 2014-06-11

**Authors:** Koichi Kobayashi, Sho Fujii, Daichi Sasaki, Shinsuke Baba, Hiroyuki Ohta, Tatsuru Masuda, Hajime Wada

**Affiliations:** ^1^Graduate School of Arts and Sciences, The University of TokyoTokyo, Japan; ^2^Center for Biological Resources and Informatics, Tokyo Institute of TechnologyYokohama, Japan; ^3^Earth-Life Science Institute, Tokyo Institute of TechnologyTokyo, Japan; ^4^Core Research for Evolutional Science and Technology, Japan Science and Technology AgencyTokyo, Japan

**Keywords:** chlorophyll, chloroplast development, digalactosyldiacylglycerol, galactolipid, monogalactosyldiacylglycerol, photosynthesis, transcriptional regulation

## Abstract

Biogenesis of thylakoid membranes in chloroplasts requires the coordinated synthesis of chlorophyll and photosynthetic proteins with the galactolipids monogalactosyldiacylglycerol (MGDG) and digalactosyldiacylglycerol (DGDG), which constitute the bulk of the thylakoid lipid matrix. MGD1 and DGD1 are the key enzymes of MGDG and DGDG synthesis, respectively. We investigated the expression profiles of *MGD1* and *DGD1* in *Arabidopsis* to identify the transcriptional regulation that coordinates galactolipid synthesis with the synthesis of chlorophyll and photosynthetic proteins during chloroplast biogenesis. The expression of both *MGD1* and *DGD1* was repressed in response to defects in chlorophyll synthesis. Moreover, these genes were downregulated by norflurazon-induced chloroplast malfunction via the GENOMES-UNCOUPLED1-mediated plastid signaling pathway. Similar to other photosynthesis-associated nuclear genes, the expression of *MGD1* and *DGD1* was induced by light, in which both cytokinin signaling and LONG HYPOCOTYL5-mediated light signaling played crucial roles. The expression of these galactolipid-synthesis genes, and particularly that of *DGD1* under continuous light, was strongly affected by the activities of the GOLDEN2-LIKE transcription factors, which are potent regulators of chlorophyll synthesis and chloroplast biogenesis. These results suggest tight transcriptional coordination of galactolipid synthesis with the formation of the photosynthetic chlorophyll–protein complexes during leaf development. Meanwhile, unlike the photosynthetic genes, the galactolipid synthesis genes were not upregulated during chloroplast biogenesis in the roots, even though the galactolipids accumulated with chlorophylls, indicating the importance of post-transcriptional regulation of galactolipid synthesis during root greening. Our data suggest that plants utilize complex regulatory mechanisms to modify galactolipid synthesis with chloroplast development during plant growth.

## INTRODUCTION

Autotrophic plant growth depends on photosynthesis, which takes place in the photosynthetic plastids, i.e., the chloroplasts. Whereas some core photosynthetic proteins are encoded in the plant plastid genome, many photosynthetic proteins, such as light-harvesting complex subunits, are encoded in the nuclear genome and targeted to chloroplasts after synthesis in the cytosol (Jarvis and López-Juez, 2013). All the genes involved in chlorophyll synthesis also reside in the angiosperm nuclear genome (Tanaka et al., 2011). At the onset of chloroplast biogenesis, enzymes involved in chlorophyll synthesis are actively expressed together with nuclear-encoded photosynthetic proteins, and are then transported to the chloroplasts where chlorophyll synthesis takes place. Because chlorophylls and their intermediates are strong photosensitizers and are phototoxic, plant cells strictly regulate their metabolism to coincide with the development of the photosynthetic machinery. In addition to modifications in enzymatic activities, transcriptional regulation plays a pivotal role in regulating chlorophyll synthesis with chloroplast functionality (Tanaka et al., 2011). When chloroplast function is severely impaired, the expression of chlorophyll synthesis genes is downregulated globally along with nuclear photosynthetic genes, through plastid-to-nucleus retrograde signaling (Strand et al., 2003; Moulin et al., 2008).

As light strongly stimulates chloroplast development, the light signaling pathway is heavily involved in transcriptional regulation of chlorophyll synthesis and formation of the photosynthetic machinery. Before light exposure, basic helix–loop–helix transcription factors, named PHYTOCHROME-INTERACTING FACTORS (PIFs), repress genes involved in chlorophyll synthesis, whereas a basic Leu zipper transcription factor, LONG-HYPOCOTYL5 (HY5), upregulates these genes, together with nuclear photosynthetic genes, on illumination (Tanaka et al., 2011). Yet other types of transcription factors, such as GOLDEN2-LIKE1 (GLK1) and GLK2, participate in the regulation of chlorophyll synthesis genes and nuclear photosynthetic genes. Deficiencies of both GLK1 and GLK2 cause a notable reduction in the expression of chlorophyll synthesis genes in leaves (Fitter et al., 2002; Waters et al., 2009), whereas overexpression of these factors induces strong expression of the chlorophyll-related genes, with ectopic chlorophyll accumulation in non-photosynthetic organs (Nakamura et al., 2009; Kobayashi et al., 2012a; Powell et al., 2012), suggesting that GLK factors are potent upregulators of these target genes. Recently, the cytokinin-responsive GATA transcription factors GATA NITRATE-INDUCIBLE CARBON-METABOLISM-INVOLVED (GNC) and CYTOKININ-RESPONSIVE GATA TRANSCRIPTION FACTOR1 (CGA1) were also reported to induce ectopic chlorophyll accumulation in hypocotyl epidermis upon overexpression (Chiang et al., 2012). In contrast to the direct regulation of the chlorophyll synthesis genes by HY5, PIFs, and GLKs, the GATA factors upregulate the genes in an indirect manner, and their regulatory pathways remain unclear (Hudson et al., 2011).

The photosynthetic chlorophyll–protein complexes are embedded in a lipid matrix of thylakoid membranes, mainly composed of the galactolipids monogalactosyldiacylglycerol (MGDG) and digalactosyldiacylglycerol (DGDG). These lipids are required not only for formation of the lipid bilayer but also for the structure and function of the photosynthetic complexes (Kobayashi et al., 2013a). Therefore, a coordinated synthesis of galactolipids with chlorophylls and photosynthetic proteins is essential for the construction of the thylakoid membrane networks. The synthesis of MGDG is catalyzed by MGDG synthase, which transfers galactose from UDP-galactose to diacylglycerol (Kobayashi et al., 2009b). Of the three MGDG synthases (MGD1, MGD2, and MGD3) in *Arabidopsis*, MGD1 is the major isoform responsible for the bulk of galactolipid synthesis (Kobayashi et al., 2007), whereas MGD2 and MGD3 constitute an alternative pathway, functioning under phosphate-deficient growth conditions (Kobayashi et al., 2009a). The expression of *MGD1* is widespread in photosynthetic tissues, whereas those of *MGD2* and *MGD3* are rarely found in green tissues under nutrient-sufficient conditions; however, *MGD2* and *MGD3* expression is strongly induced under phosphate deficient conditions (Awai et al., 2001; Kobayashi et al., 2004). Not only is MGDG the main constituent of thylakoid membranes, it is also a substrate for DGDG synthesis. DGDG is predominantly synthesized by the addition of a second galactose from UDP-galactose to MGDG, which is catalyzed by DGDG synthase (Kelly and Dörmann, 2002; Kelly et al., 2003). Higher plants, including *Arabidopsis*, also have paralogous genes for DGDG synthase, namely *DGD1* and *DGD2* (Gaude et al., 2004). Analysis of *Arabidopsis* mutants has shown that the bulk of DGDG found in chloroplasts is synthesized by DGD1 in combination with MGD1 (Dörmann et al., 1995).

Recently we reported that a defect in galactolipid synthesis leads to strong downregulation of chlorophyll synthesis and photosynthetic genes, suggesting a coupled regulation between galactolipid synthesis and the formation of photosynthetic chlorophyll–protein complexes (Kobayashi et al., 2013a). Considering that galactolipids are essential components of thylakoid membranes and photosynthesis, their synthesis is expected to be regulated alongside that of chlorophylls and photosynthetic proteins. It has been reported that the activity of MGD1 is post-translationally controlled by a redox-dependent oxidation/reduction (Yamaryo et al., 2006; Shimojima et al., 2013) and by dissociation/association of phosphatidic acid (Dubots et al., 2010; Shimojima et al., 2013). On the other hand, there is only limited data on the transcriptional regulation of galactolipid synthesis during chloroplast biogenesis (Kobayashi et al., 2009b), in contrast to the in-depth insights into the regulatory mechanisms of the genes involved in chlorophyll synthesis and photosynthesis (Tanaka et al., 2011; Jarvis and López-Juez, 2013). In this study, we investigated the relationships among the expression of galactolipid synthesis genes and chlorophyll synthesis, chloroplast functionality, light and cytokinin signaling, and several chloroplast-related transcription factors.

## MATERIALS AND METHODS

### PLANT MATERIALS AND GROWTH CONDITIONS

All plants used in this study were the Columbia ecotype of *Arabidopsis thaliana*. The *cs* (Koncz et al., 1990), *chli1*, *chlh* (Huang and Li, 2009), *hema1* (Woodson et al., 2011), *genomes-uncoupled* (*gun*)* 1-1*, *pOCA107-2* (the parental line for the *gun* mutants; Susek et al., 1993), *gun4-1*, *gun5-1* (Mochizuki et al., 2001), *hy5-215* (Oyama et al., 1997), *ahk2-2 ahk3-3* (Higuchi et al., 2004), *glk1 glk2* (Fitter et al., 2002), *GLK1ox* (*35S:GLK1*), *GLK2ox* (*35S:GLK2*; Waters et al., 2008), *gnc cga1* (Chiang et al., 2012), and *slr-1* (Fukaki et al., 2002) mutants were described previously. Plants were grown on Murashige and Skoog (MS) medium (adjusted to pH 5.7 with KOH) containing 1.0% (w/v) sucrose solidified with 0.8% (w/v) agar in plates at 23°C under continuous white light (40 μmol photons m^-^^2^ s^-^^1^), with the exception of the experiment in **Figure 3**. For the analysis in **Figure 3**, sterilized seeds were sown in flasks with 20 mL liquid MS media. Each flask was completely wrapped with aluminum foil after 3 h of light exposure and incubated on the rotary shaker at 22°C. After growth for 4 days in the dark, etiolated seedlings were treated with white light (40 μmol photons m^-^^2^ s^-^^1^) or with 1 μM 6-benzyladenine (BA) for the indicated period. For the norflurazon (NF) treatment in **Figure 2**, plants were germinated and grown in MS medium containing 1 μM NF. For the analysis of roots, plants were grown vertically in the MS medium for the indicated period, and roots were detached from the shoot at the root–hypocotyl junction for sampling. For the BA treatment in **Figure 5A**, 7-day-old wild-type seedlings were transferred to the MS medium containing 1 μM BA and grown for another 7 days. All plants were grown in a growth chamber (CLE-303, Tomy Seiko, Tokyo, Japan).

### CHLOROPHYLL DETERMINATION

Whole seedlings were pulverized in liquid nitrogen, homogenized in 80% (v/v) acetone, and debris was removed by centrifugation at 10,000 × *g* for 5 min. The absorbances of the supernatant at 720 nm, 663 nm, 647 nm, and 645 nm were measured using an Ultrospec 2100 *pro* spectrophotometer (GE Healthcare Bioscience, Amersham, UK). The chlorophyll (*a* and* b*) concentration of the samples was determined as described in Melis et al. (1987).

### GENE EXPRESSION ANALYSIS

Gene expression was examined by a real-time quantitative RT-PCR (Q-PCR) analysis (Pfaffl, 2001). Total RNA was extracted using the RNeasy Plant Mini kit (Qiagen, Hilden, Germany). Genomic DNA digestion and reverse transcription were performed using the PrimeScript RT Reagent kit with gDNA Eraser (TaKaRa Bio, Otsu, Japan) according to the manufacturer’s instructions. cDNA amplification was performed using the Thunderbird PreMix kit (Toyobo, Osaka, Japan) and 200 nM of the following gene-specific primers: *MGD1* (AT4G31780): forward primer, 5′-GCAGGACTTGAAACATCACAAATC-3′; reverse primer, 5′-GCGAACTGGTTTCACAAAGGA-3′; *DGD1* (AT3G11670): forward primer, 5′-CTGAAGAGAGATCCCGTGGTG-3′; reverse primer, 5′-TCCCAAGTTCGCTTTTGTGTT-3′; *DGD2* (AT4G00550): forward primer, 5′-TGCAGAACCTATGACGATGGA-3′; reverse primer, 5′-GCTCTGTAAGTTGCGATGGTTG-3′; *LIGHT HARVESTING COMPLEX PHOTOSYSTEM II SUBUNIT 6* (*LHCB6*, AT1G15820): forward primer, 5′-GGACTTTGAGAAGCTGGAGAGG-3′; reverse primer, 5′-ACAAACCAAGAGCACCGAGAG-3′; *H SUBUNIT OF MG-CHELATASE* (*CHLH*, AT5G13630): forward primer, 5′-TGGTAGAGAGACAGAAGCTCGAAA-3′; reverse primer, 5′-CCAAAGAACCTGCCCAAGAG-3′; *ACTIN8* (AT1G49240): forward primer, 5′-ACTGTGCCTATCTACGAGGGTTTC-3′; reverse primer, 5′-CCCGTTCTGCTGTTGTGGT-3′. The amplification efficiency with each primer set was determined with a dilution series of the cDNA pool from wild-type samples. No or negligible signals were detected in no-template control with each primer set. Thermal cycling consisted of an initial denaturation step at 95°C for 10 s, followed by 40 cycles of 95°C for 5 s and 60°C for 30 s. Melting curve runs were performed at the end of each PCR to verify the specificity of the primers. Signal detection and quantification were performed in duplicate using a MiniOpticon (Bio-Rad, Hercules, CA, USA). The relative abundance of all transcripts amplified was normalized to the constitutive expression level of *ACTIN8* according to the efficiency correction method (Pfaffl, 2001). Three independent biological experiments were performed for each sample.

### LIPID AND FATTY ACID ANALYSES

Total lipids were extracted from roots pulverized in liquid nitrogen and were separated by thin-layer chromatography using a solvent system of acetone/toluene/water (136:45:12, v/v/v) as described by Dörmann et al. (1995) and Yamaryo et al. (2003). Lipids were visualized with 0.01% (w/v) primuline in 80% (v/v) acetone under UV light. MGDG, DGDG, and a mixture of other glycerolipids, including phosphatidylcholine, phosphatidylethanolamine, phosphatidylinositol, phosphatidylglycerol, and sulfoquinovosyldiacylglycerol, were isolated from silica gel plates. Fatty acids in each lipid fraction were methyl-esterified by incubating with a 5% (v/v) HCl in methanol solution at 85°C for 2 h and quantified by gas chromatography (GC-17, Shimadzu, Kyoto, Japan) using myristic acid as an internal standard.

### STATISTICAL ANALYSIS

Two-tailed Student’s *t*-test with equal variance was performed using Microsoft Excel 2010 for Windows.

## RESULTS

### THE INFLUENCE OF CHLOROPHYLL SYNTHESIS ON *MGD1* AND *DGD1* EXPRESSION

**FIGURE 1:**
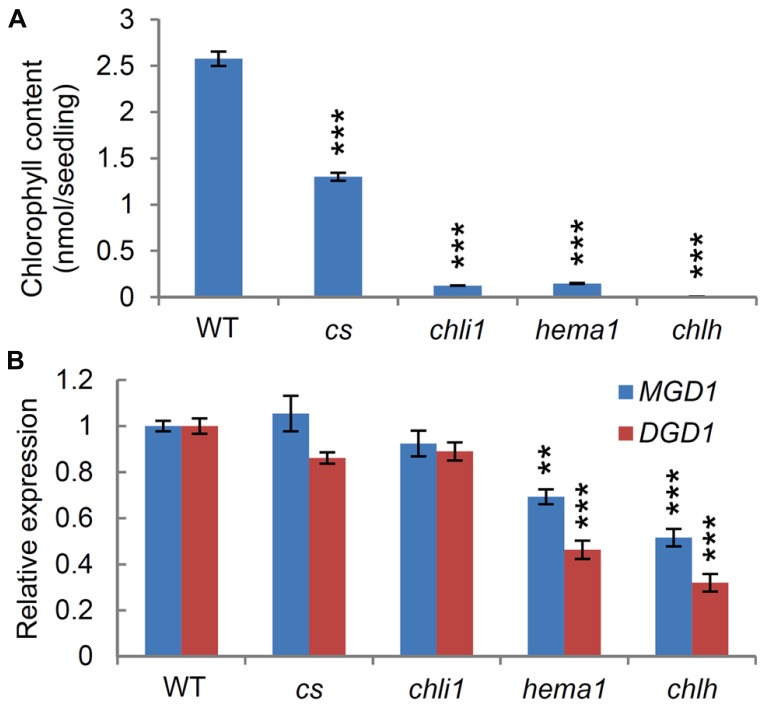
**Effects of chlorophyll synthesis on the expression of *MGD1* and *DGD1*. (A)** Chlorophyll contents in 7-day-old wild type (WT) and mutants deficient in chlorophyll synthesis. **(B)** Quantitative RT-PCR analysis of *MGD1* and *DGD1* in 7-day-old chlorophyll mutants. Values are presented as the fold difference from the WT after normalizing to the control gene *ACTIN8*. Data shown in **(A) **and **(B)** are the mean ± SE from three independent experiments. Asterisks indicate a significant difference from the wild-type (***P < 0.001, **P < 0.01, Student’s *t*-test).

To address whether chlorophyll metabolism influences expression of galactolipid synthesis genes, we examined the expression levels of *MGD1* and *DGD1* in chlorophyll-deficient mutants. *chlh*, *chli1* (Huang and Li, 2009), and *hema1* (Woodson et al., 2011) are knockout mutants for *CHLH*, *CHLI1*, and *HEMA1*, respectively, whereas the *cs* mutant (Koncz et al., 1990) is a knockdown mutant for *CHLI1*. *HEMA1* encodes the major isoform of glutamyl-tRNA reductase, catalyzing the synthesis of 5-aminolevulinic acid (the first committed step of tetrapyrrole synthesis; Tanaka et al., 2011). *CHLH* is a single gene encoding the subunit H of magnesium chelatase, whereas *CHLI1* encodes the major isoform of the two paralogs of magnesium chelatase subunit I in *Arabidopsis*. We compared chlorophyll contents in 7-day-old seedlings among the mutants and wild-type seedlings (**Figure 1A**). As reported previously, the *chlh* mutant did not contain any chlorophyll (Huang and Li, 2009), whereas the *cs* mutant contained half the level of chlorophyll present in the wild-type (Rissler et al., 2002; Kobayashi et al., 2008). The *hema1* and *chli1* mutants had 95% less chlorophyll than the wild-type seedlings, consistent with previous reports (Huang and Li, 2009; Kobayashi et al., 2013a). Q-PCR analyses revealed that the *chlh* mutant had the largest decrease in *MGD1* and *DGD1* expression, suggesting that a complete loss of chlorophyll synthesis induces downregulation of the major galactolipid synthesis genes. There was also a significant decrease in *MGD1* and *DGD1* expression in the *hema1* mutant. Although total chlorophyll levels were equivalent in *chli1* and *hema1* mutants, the expression levels of *MGD1* and *DGD1* in *chli1* were higher than those in *hema1*, and were almost comparable to the wild-type. In addition, no significant differences in the expression levels of the galactolipid synthesis genes were observed between the wild-type and the *cs* mutant. As HEMA1 operates within a pathway that is also used for tetrapyrrole, heme, and chlorophyll synthesis (Tanaka et al., 2011), the *hema1* mutation could have a greater effect than the *chil1* mutation, which might result in stronger repression of *MGD1* and *DGD1*. Our data suggest that it is not simply the level of chlorophyll synthesis that influences the expression of the major galactolipid synthesis genes, but also the functional state of tetrapyrrole and/or chloroplast metabolism.

### THE INVOLVEMENT OF RETROGRADE SIGNALING IN *MGD1* AND *DGD1* EXPRESSION

The reduced expression of *MGD1* and *DGD1* in *chlh* and *hema1* suggests a repression mechanism of these genes in response to an impairment of tetrapyrrole synthesis or chloroplast function. It is known that photosynthesis-associated nuclear genes are downregulated through signals transmitted retrogradely from plastids (plastid signals) when chloroplast functions are severely disrupted (Tanaka et al., 2011; Jarvis and López-Juez, 2013). To assess whether plastid signals are involved in the regulation of galactolipid synthesis genes, we examined expression levels of *MGD1* and *DGD1* in *gun* mutants (*gun1*, *gun4*, and *gun5*), which are deficient in plastid signaling (Susek et al., 1993; Mochizuki et al., 2001). The *pOCA107-2* line, which is the parental line of the *gun* mutants (Susek et al., 1993), was used as the wild-type control.

**FIGURE 2:**
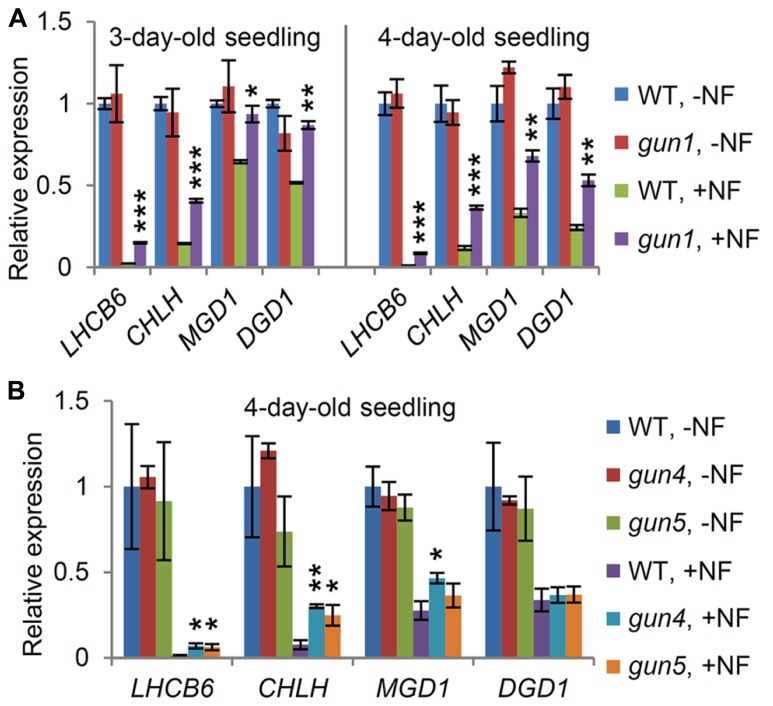
**Involvement of plastid signaling in the expression of *MGD1* and *DGD1*.** Quantitative RT-PCR analysis of *MGD1*, *DGD1*, *LHCB6*, and *CHLH* in **(A)** the *gun1* and **(B)** *gun4* and *gun5* seedlings grown with (+NF) or without norflurazon (-NF) for 3 or 4 days. The parental *pOCA107-2* line was used as the wild-type (WT) control. Values are presented as the fold difference from untreated WT (WT, –NF) after normalizing to the control gene *ACTIN8*. Data shown are the mean ± SE from three independent experiments. Asterisks indicate a significant difference from the wild-type in each +NF or –NF condition (***P < 0.001, **P < 0.01, *P < 0.05, Student’s *t*-test).

Plants were germinated and grown for 3 days (**Figure 2A**, left) or 4 days (**Figure 2A**, right) in the presence or absence of NF, which inhibits carotenoid synthesis and, consequently, chloroplast functions. As reported for many other photosynthesis-associated nuclear genes (Strand et al., 2003), expression levels of *LHCB6* and* CHLH* were substantially decreased by NF treatment in the wild-type seedlings (**Figure 2A**). *MGD1* and *DGD1* expression also decreased in the 3-day-old wild type after NF treatment, although not as much as *LHCB6* and* CHLH*. In the 4-day-old wild type, *MGD1* and *DGD1* expression was more strongly repressed than in the 3-day-old wild-type, suggesting that a longer NF treatment led to stronger downregulation of these genes. In the *gun1* mutant, expression levels of all these genes did not differ from those in the wild-type in the absence of NF. Meanwhile, *LHCB6* and* CHLH* expression in the NF-treated *gun1* mutant was higher than that in the NF-treated wild type, consistent with previous reports (Susek et al., 1993; Moulin et al., 2008). Moreover, the 3-day-old *gun1* seedlings showed no obvious decrease in *MGD1* and *DGD1* expression upon NF treatment. Even in the 4-day-old *gun1* seedlings, higher expression of *MGD1* and *DGD1* was observed compared with that in wild-type. These results suggest that GUN1 plays a pivotal role in the downregulation of the major galactolipid synthesis genes when chloroplast functions are severely impaired.

To ascertain a possible involvement of plastid signaling through the chlorophyll biosynthetic pathway in the downregulation of *MGD1* and *DGD1*, we investigated *gun4* and *gun5* mutants, which have a *gun* phenotype due to mutations in the *GUN4* and the *CHLH* genes, respectively (Mochizuki et al., 2001; Larkin et al., 2003). In this experiment, we grew seedlings in the presence or absence of NF for 4 days after planting. In the absence of NF, both *gun4* and *gun5* showed no decrease in *MGD1* and *DGD1* expression compared with the wild-type (**Figure 2B**), despite having reduced chlorophyll in the leaves (Mochizuki et al., 2001). The results agree with the data in **Figure 1**, showing that a slight decrease in chlorophyll synthesis does not influence the expression of the galactolipid synthesis genes. In the presence of NF, however, *LHCB6* and* CHLH* expression in the *gun4* and *gun5* mutants was substantially higher than that in the wild-type control, consistent with previous reports (Strand et al., 2003; Moulin et al., 2008). Meanwhile, unlike in *gun1*, the expression levels of *MGD1* and *DGD1* in the *gun4* and *gun5* mutants were not largely different from those in the wild-type, reflecting a minor impact of chlorophyll metabolism-mediated plastid signaling on the downregulation of the galactolipid synthesis genes.

### TRANSCRIPTIONAL REGULATION OF GALACTOLIPID SYNTHESIS BY LIGHT AND CYTOKININ DURING PHOTOMORPHOGENESIS

**FIGURE 3:**
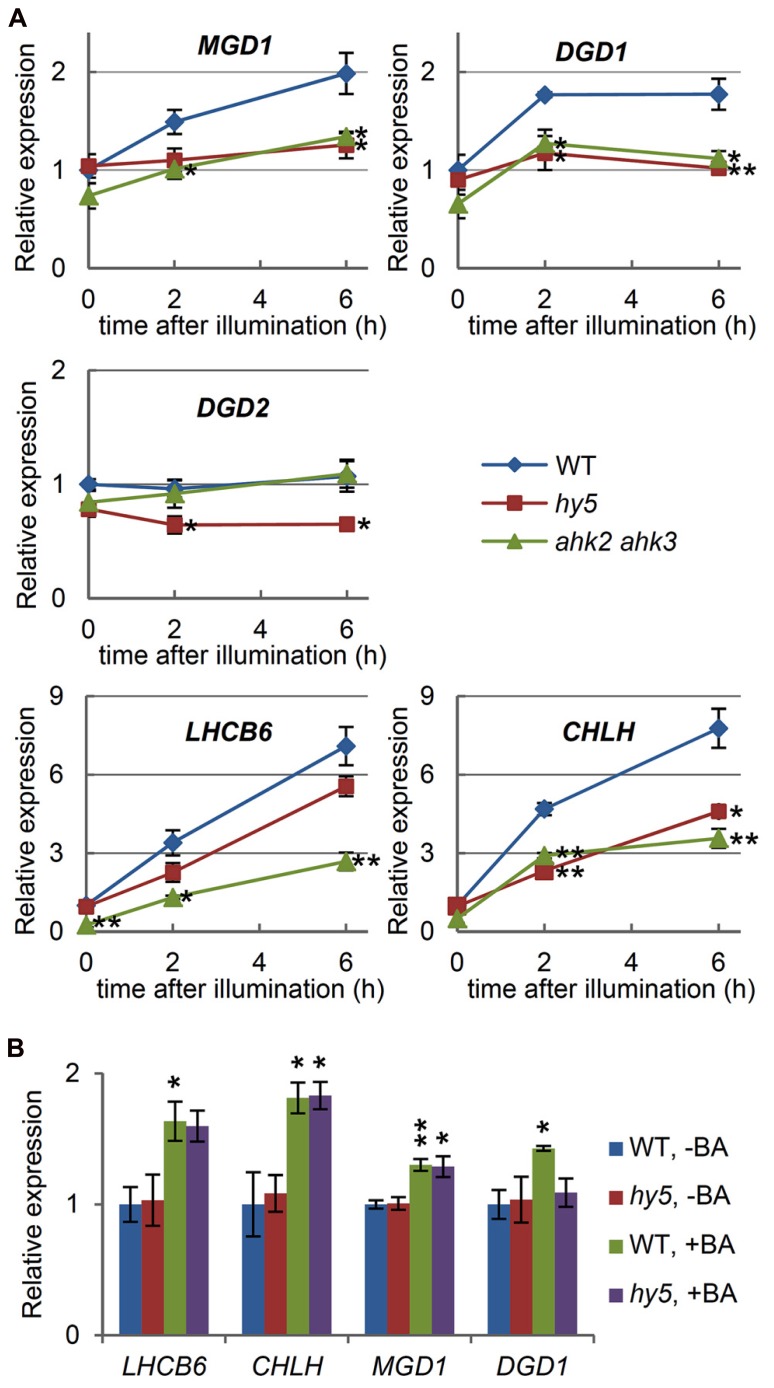
**Involvement of light and cytokinin signaling in the expression of *MGD1* and *DGD1*. (A)** Quantitative RT-PCR analysis of galactolipid synthesis genes (*MGD1*, *DGD1*, and *DGD2*) and chlorophyll-related genes (*LHCB6* and *CHLH*) in wild-type (WT), the *hy5* mutant and the *ahk2 ahk3* double mutant after illumination of 4-day-old etiolated seedlings for the indicated times. **(B)** Quantitative RT-PCR analysis in 4-day-old etiolated seedlings of WT and the *hy5* mutant treated with 6-benzyladenine (+BA) or without BA (–BA) for 6 h. Values are presented as the fold difference relative to **(A)** the unilluminated seedlings (0 h) or **(B)** the untreated wild-type control (WT, –BA) after normalizing to the control gene *ACTIN8*. Data shown are the mean ± SE from three independent experiments. Asterisks indicate a significant difference **(A)** from the wild-type at each time point and **(B)** from the BA-untreated samples of each plant (**P < 0.01, *P < 0.05, Student’s *t*-test).

Light plays a pivotal role in triggering chloroplast biogenesis, including galactolipid and chlorophyll synthesis during photomorphogenesis. We reported previously that *MGD1*, but not *MGD2* or *MGD3*, is upregulated by light (Kobayashi et al., 2004). Consistent with these results, *MGD1* expression in the wild-type increased twofold after illumination of etiolated seedlings for 6 h (**Figure 3A**). No light induction analysis has been reported for *DGD* genes, although mutant analyses demonstrate that DGD1 is mainly responsible for DGDG synthesis in photosynthetic tissues (Kelly et al., 2003). Investigation of the light responses of the *DGD* genes in the wild-type seedlings showed that *DGD1* expression had increased upon illumination, whereas *DGD2* expression remained unchanged (**Figure 3A**). This result supports the major and minor roles of *DGD1* and *DGD2*, respectively, for thylakoid biogenesis during the development of photosynthetic tissues (Kelly et al., 2003). Light-induced upregulation of *LHCB6* and* CHLH* was also observed. The increases of *LHCB6* and* CHLH* expression were more pronounced than those of *MGD1* and *DGD1*, in line with the extremely large accumulation of chlorophyll compared with the smaller increase in galactolipids during photomorphogenesis (Poincelot, 1973; Selstam and Sandelius, 1984).

It has been reported that transcription factor HY5, which is a positive regulator of photomorphogenesis downstream of multiple photoreceptors, directly targets many of the light-inducible photosynthesis-associated genes, including key chlorophyll synthesis genes (Lee et al., 2007). Indeed, upon illumination, the upregulation of *CHLH*, which is a target of HY5, was strongly reduced in a null *hy5* mutant (*hy5-215*; **Figure 3A**). In contrast, a deficiency of HY5 only weakly influenced *LHCB6* expression, consistent with reports that *LHCB6* may not be a direct target of HY5 (Lee et al., 2007). It is interesting to note that among the galactolipid synthesis genes, *DGD1* and *DGD2* are putative direct targets of HY5 (Lee et al., 2007). In fact, the expression of these genes under light conditions was reduced in the *hy5* seedlings compared with the wild-type (**Figure 3A**). Moreover, we observed that light-induced upregulation of *MGD1* did not occur in *hy5*, even though *MGD1* was not perceived as a HY5 target. Our data suggest that HY5 plays a role in the light-induction of genes involved in galactolipid and chlorophyll synthesis, both directly and indirectly.

In addition to light, cytokinin has been reported to stimulate expression of *MGD1* in etiolated seedlings (Kobayashi et al., 2004). To assess the involvement of cytokinin signaling in the light-response of galactolipid synthesis genes, a double mutant for cytokinin receptors (*ahk2 ahk3*; Higuchi et al., 2004) was investigated. Under dark conditions, the expression of both *MGD1* and *DGD1* decreased by ~30% in *ahk2 ahk3* compared with the wild-type, although the differences were not significant (**Figure 3A**). The expression of *LHCB6* and* CHLH* under dark conditions also decreased in *ahk2 ahk3*. The data may reflect a minor role for cytokinin signaling in the expression of these genes during skotomorphogenesis. Furthermore, light induction of *MGD1* and *DGD1* expression did not occur in the double mutant. A similar result was observed for *LHCB6* and* CHLH* expression, whereas *DGD2* expression was not different between the wild-type or the *ahk2 ahk3* mutants under light conditions. Our data demonstrate that cytokinin signaling is involved in a coordinated upregulation of the light-responsive genes during photomorphogenesis.

Exogenous cytokinin treatment is known to induce chloroplast development and to activate light-regulated promoters under dark conditions (Chory et al., 1994). In our experiments, expression of *LHCB6* and* CHLH* under dark conditions was significantly upregulated after treatment with 1 μM BA, a synthetic cytokinin (**Figure 3B**), which is consistent with the findings that cytokinin stimulates chlorophyll synthesis in etiolated seedlings (Masuda et al., 1995; Hedtke et al., 2012). As reported previously (Kobayashi et al., 2004), *MGD1* expression was also significantly upregulated by BA treatment under dark conditions. In addition, *DGD1* expression increased in response to BA, supporting the results obtained from the *ahk2 ahk3* mutant that cytokinin signaling has a positive effect on the expression of these genes. Since it has been reported that cytokinin can promote the expression of anthocyanin biosynthetic genes through stabilization of the HY5 protein (Vandenbussche et al., 2007), we investigated the involvement of HY5 in cytokinin signaling. When the *hy5* seedlings were treated with 1 μM BA under dark conditions, *MGD1* was upregulated to almost the same level as that in the BA-treated wild-type seedlings (**Figure 3B**). *LHCB6* and* CHLH* expression in the BA-treated *hy5* seedlings was also equivalent to the BA-treated wild-type seedlings, suggesting that BA can upregulate these genes independently of HY5. In contrast, the increase in *DGD1* expression by BA appeared to be canceled in *hy5*. Thus, the regulatory mechanisms may differ between *MGD1* and *DGD1*, as represented by the direct and non-direct targeting by HY5.

### HOMEOSTATIC TRANSCRIPTIONAL REGULATION OF GALACTOLIPID BIOSYNTHESIS

**FIGURE 4:**
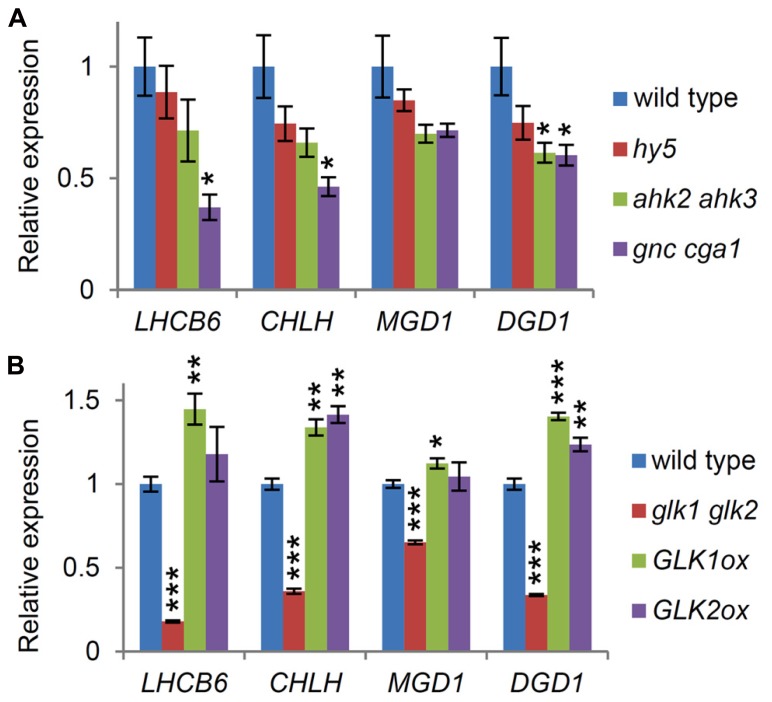
**Involvement of transcription factors associated with chloroplast development in the expression of *MGD1* and *DGD1*.** Quantitative RT-PCR analysis of *MGD1*, *DGD1*, *LHCB6*, and *CHLH* in seedlings grown for 7 days under continuous light in both **(A)** and **(B)**. Values are presented as the fold difference from the wild-type after normalizing to the control gene *ACTIN8*. Data shown are the mean ± SE from three independent experiments. Asterisks indicate significant difference from the wild-type (***P < 0.001, **P < 0.01, *P < 0.05, Student’s *t*-test).

Genes for the major galactolipid synthesis pathway (*MGD1* and *DGD1*), but not those for the alternative pathway (*MGD2*, *MGD3*, and *DGD2*), are upregulated along with photosynthesis-associated genes in response to light (**Figure 3A**; Kobayashi et al., 2004), consistent with the crucial roles of the major pathway in thylakoid biogenesis and photosynthesis (Kobayashi et al., 2009b). To assess the importance of light and cytokinin signaling pathways for homeostatic gene expression in galactolipid synthesis during early seedling growth, we analyzed the expression of *MGD1* and *DGD1* and that of *LHCB6* and *CHLH* in the *hy5* and *ahk2 ahk3* mutants, which were grown for 7 days under continuous white light. Although the expression of these genes appeared to decrease in the mutants compared with the wild-type, only the reduction in *DGD1* expression in *ahk2 ahk3* was statistically significant (**Figure 4A**), suggesting a reduced importance of HY5 and cytokinin signaling in the homeostatic expression of these genes under continuous light conditions.

We assessed whether other factors associated with chloroplast biogenesis were involved in the regulation of the galactolipid synthesis genes. In this study, we examined the involvement of two types of transcription factors, namely GATA factors (GNC and CGA1) and GLK factors (GLK1 and GLK2), in the expression of *MGD1* and *DGD1*. In the 7-day-old *gnc cga1* double mutant, the expression of both *LHCB6* and *CHLH* was less than half that in the wild-type (**Figure 4A**), consistent with the reduced chlorophyll contents in mutants deficient in these factors in various tissues and conditions (Hudson et al., 2011; Chiang et al., 2012). *DGD1* expression levels also decreased significantly in the mutant, whereas the decrease in *MGD1* expression was not statistically significant. These data indicate that GNC and CGA1 are involved in the expression of the galactolipid synthesis genes, but that their influences are marginal.

Next we analyzed the expression levels of these genes in a double mutant (*glk1 glk2*) and in overexpressors of the GLK factors (*GLK1ox* and *GLK2ox*). As reported previously (Waters et al., 2009), the expression of *LHCB6* and *CHLH*, known targets of GLKs, decreased markedly in the *glk1 glk2* mutant, whereas their expression levels increased slightly in the GLK overexpressors (**Figure 4B**). Interestingly, *DGD1* showed an expression pattern similar to that of *LHCB6* and *CHLH* in these plants, although *DGD1* was not a reported target of the GLK factors (Waters et al., 2009). *MGD1* expression also decreased significantly in the *glk1 glk2* mutant, but the decrease was less than those of other genes examined. Furthermore, no significant upregulation of *MGD1* was observed in either *GLK1ox* or *GLK2ox*, indicating a minor involvement of GLK factors in the regulation of *MGD1* expression compared with *DGD1* expression. As discussed later, smaller reductions in *MGD1* expression than those in *DGD1* in these mutants may reflect differences in mode of regulation for each step; *DGD1* is subject to strong transcriptional regulation, whereas *MGD1* may be rather under posttranscriptional regulation.

### REGULATION OF GALACTOLIPID METABOLISM DURING CHLOROPLAST DIFFERENTIATION IN THE ROOT

**FIGURE 5:**
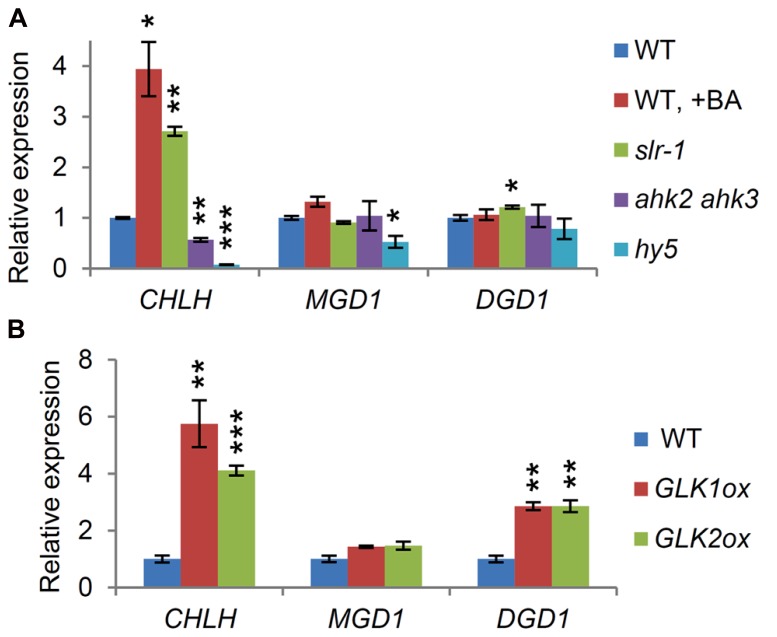
**Regulation of *MGD1* and *DGD1* expression in the root.** Quantitative RT-PCR analysis of *CHLH*, *MGD1* and *DGD1* in roots of **(A)** 14-day-old seedlings and **(B)** 21-day-old seedlings grown under continuous white light. Values are presented as the fold difference from the untreated wild-type control (WT) after normalizing to the control gene *ACTIN8*. Data shown are the mean ± SE from three independent experiments. +BA, 6-benzyladenine treatment for 7 days. Asterisks indicate significant difference from the wild-type control (***P < 0.001, **P < 0.01, *P < 0.05, Student’s* t*-test).

We recently reported that chloroplast development in the roots is regulated through auxin/cytokinin signaling, involving at least two transcription factors, HY5 and GLK2 (Kobayashi et al., 2012a). In fact, seedlings treated with BA, or auxin-signaling mutants, like the *slr-1* mutant, accumulated chlorophyll in the roots and displayed an upregulation in chlorophyll synthesis genes, such as *CHLH* (Kobayashi et al., 2012a). Meanwhile, the roots of the *hy5* and the *ahk2 ahk3* double mutant showed reductions in chlorophyll synthesis gene expression and chlorophyll accumulation. Because the expression of *MGD1* and *DGD1* had a pattern similar to that of *CHLH* during photomorphogenesis (**Figure 3**), we examined whether *MGD1* and *DGD1* were upregulated together with *CHLH* during chloroplast differentiation in the roots. Whereas *CHLH* expression in the roots increased in the *slr-1* mutant and the BA-treated wild-type and decreased in *hy5* and *ahk2 ahk3* as reported previously (Kobayashi et al., 2012a), the expression of *MGD1* and *DGD1* in these root samples remained at levels similar to that in the untreated wild-type, except for some slight, but significant, changes (**Figure 5A**). This result indicates an uncoordinated regulation of gene expression between chlorophyll synthesis and galactolipid synthesis during chloroplast development in the root.

We recently reported that GLK factors strongly induce chloroplast development with an upregulation of photosynthesis-associated genes in the root (Kobayashi et al., 2012a, 2013b). To address whether the excessive chlorophyll synthesis accompanied upregulation of galactolipid synthesis genes in the roots, we examined the expression levels of *MGD1* and *DGD1* in the roots of the GLK overexpressors. The results showed that *DGD1*, but not *MGD1*, was strongly upregulated together with *CHLH* in *GLK1ox* and* GLK2ox* roots (**Figure 5B**).

**FIGURE 6:**
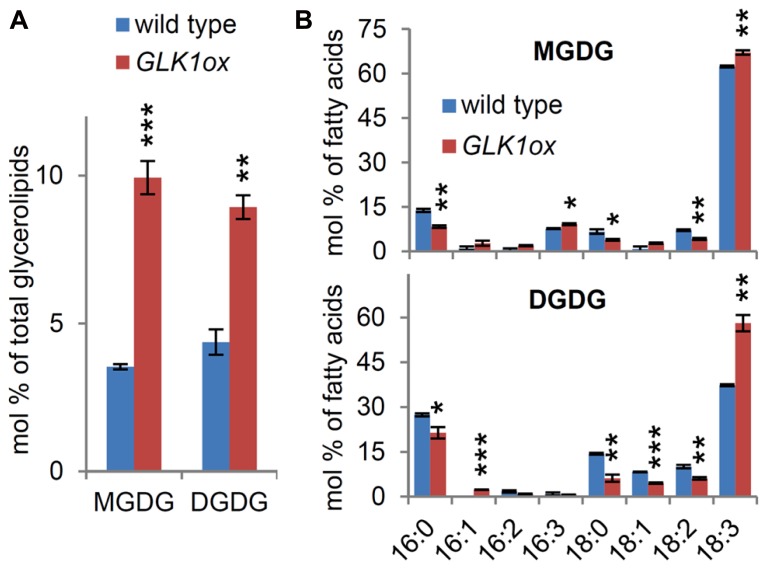
**Galactolipid analysis in the roots of wild-type and the *GLK1ox* transgenic plants. (A)** Galactolipid contents and **(B)** fatty acid composition of the lipids in roots of 21-day-old seedlings grown under continuous light. Values are the means ± SE from three independent experiments. Asterisks indicate significant difference from the wild-type (***P < 0.001, **P < 0.01, *P < 0.05, Student’s *t*-test).

To assess whether *MGD1* and *DGD1* expression was coupled with lipid homeostasis during chloroplast differentiation, we compared galactolipid contents in the roots between the wild-type and *GLK1ox*. In the *GLK1ox* root, the proportions of MGDG and DGDG to total membrane glycerolipids increased more than twofold compared with those in the wild-type root (**Figure 6A**), showing that GLK1-mediated chloroplast differentiation accompanies the accumulation of galactolipids without upregulation of *MGD1* expression in the root. When the fatty acid composition of MGDG in the *GLK1ox* root was compared with that in the wild-type root, the proportions of triunsaturated fatty acids (16:3 and 18:3) increased in the *GLK1ox* root (**Figure 6B**). The increase in the 18:3 fatty acids was also observed in DGDG of the *GLK1ox* root, suggesting activation of a leaf-type galactolipid metabolism in the root plastids (Kobayashi et al., 2009b). The accumulation of galactolipids is consistent with the prominent development of thylakoid membrane networks within the plastids in the *GLK1ox* root (Kobayashi et al., 2013b). Because fatty acid desaturation in plastids requires ferredoxins as electron donors and is closely linked with the photosynthetic activities (Shanklin and Cahoon, 1998), development of thylakoid membranes and photosynthetic machineries may affect desaturase activities in plastids and thereby alter fatty acid compositions of galactolipids in *GLK1ox* roots. It is also possible that *GLK1ox* upregulates genes involved in fatty acid desaturation in roots.

## DISCUSSION

### TRANSCRIPTIONAL COORDINATION OF GALACTOLIPID SYNTHESIS WITH CHLOROPHYLL SYNTHESIS AND CHLOROPLAST DEVELOPMENT

In our previous study, we reported that galactolipid biosynthetic activities profoundly influence gene expression related to photosynthesis and chlorophyll synthesis; a lack of galactolipid synthesis in the *mgd1-2* mutant caused a marked downregulation of the photosynthesis-associated genes, whereas partial complementation of galactolipid synthesis in *mgd1-2* by the alternative MGD2/MGD3 pathway attenuated the downregulation of these genes (Kobayashi et al., 2013a). In this study, we revealed that tetrapyrrole metabolism also affected the expression of galactolipid synthesis genes, as observed by the downregulation of *MGD1* and *DGD1* in the *chlh* and *hema1* mutants (**Figure 1B**). Taken together, our data suggest a strong transcriptional link between galactolipid and chlorophyll synthesis, which would be required for the coordinated formation of lipid bilayers, with chlorophyll synthesis and the assembly of photosynthetic complexes during thylakoid biogenesis. One possible mechanism that coordinates the expression of the galactolipid synthesis genes with that of the photosynthesis-associated genes is plastid signaling. Indeed, as with many photosynthesis-associated genes, *MGD1* and *DGD1* expression was regulated by plastid signaling, in which GUN1 played a pivotal role (**Figure 2A**). It is possible that in *chlh* and *hema1*, impairments in chloroplast function due to disruption of tetrapyrrole metabolism, induce plastid signaling via GUN1, which downregulates the galactolipid synthesis genes. It is interesting to note that *MGD1* and *DGD1* expression was not repressed in the *chli1* mutant, although the chlorophyll deficiency in *chli1* was almost the same as in *hema1* (**Figure 1**), suggesting that the inability to accumulate chlorophyll does not simply trigger downregulation of the galactolipid synthesis genes. It has recently been reported that increased flux through the heme branch of the tetrapyrrole biosynthetic pathway restores the expression of photosynthesis-associated nuclear genes under chloroplast-defective conditions, suggesting that heme synthesized in plastids may be used as a retrograde signal to upregulate nuclear photosynthetic genes (Woodson et al., 2011). Thus, it is possible that changes in heme metabolism would also contribute to the downregulation of the galactolipid synthesis genes in *hema1*. In the *chlh* mutant, the complete lack of chlorophyll synthesis may largely disrupt tetrapyrrole metabolism or lead to secondary effects in chloroplast functioning that induce downregulation of the galactolipid synthesis genes. On the other hand, in the *chli1* mutant, the CHLI2 isoform can partially complement the loss of CHLI1 in the chelation of Mg to protoporphyrin IX (Kobayashi et al., 2008; Huang and Li, 2009) and thus the remaining activity by CHLI2 might maintain *MGD1* and *DGD1* expression at wild-type levels in *chli1*.

### COEXPRESSION OF THE MAJOR GALACTOLIPID SYNTHESIS GENES WITH PHOTOSYNTHESIS-ASSOCIATED NUCLEAR GENES

When chloroplasts are differentiated from proplastids or etioplasts under light conditions, large amounts of galactolipids are synthesized, together with chlorophylls and photosynthetic proteins, to form functional thylakoid membrane networks. Consistent with the bulk of galactolipids in thylakoid membranes being synthesized via the MGD1–DGD1 pathway (Kobayashi et al., 2009b), the expression of both *MGD1* and *DGD1* was induced by light during photomorphogenesis. In leaves, the expression patterns of these galactolipid synthesis genes were generally similar to those of *LHCB6* and *CHLH* in various mutants and under various growth conditions, although the amplitude of the expression changes were different (**Figures 2–4**), suggesting the existence of co-regulatory mechanisms between the galactolipid synthesis genes and the chlorophyll-related genes. It has been reported that key chlorophyll synthesis genes, including *CHLH*, form tight coexpression networks with other photosynthesis-associated nuclear genes, like *LHCB6*, suggesting that the regulatory coexpression in the nucleus plays a central role in the assembly of the photosynthetic machinery during chloroplast biogenesis (Kobayashi et al., 2012b). In our results, *MGD1* and *DGD1* showed a pattern of coexpression with *LHCB6 and CHLH*; however, these galactolipid synthesis genes are not found in the coexpression networks of the photosynthesis-associated genes. Because galactolipids are predominant not only in the thylakoid membranes of chloroplasts but also in the envelope membranes of plastids (Block et al., 1983), it is reasonable to assume that the expression profiles of the galactolipid synthesis genes differ from those of the photosynthetic genes, particularly in non-photosynthetic organs, such as the roots (**Figure 5**). Furthermore, additional unique functions other than photosynthesis have been proposed for galactolipids under several growth conditions, including phosphate starvation (Kobayashi et al., 2009b), wounding stress (Nakashima et al., 2013), and cold (Moellering et al., 2010). In fact, *MGD1* expression is highly upregulated by salt and drought stress in roots and by wounding in leaves (Kobayashi et al., 2009b), whereas *DGD1* expression is upregulated in response to limitations in phosphate and nitrogen (Gaude et al., 2007). This unique regulation of *MGD1* and *DGD1* may result in the removal of the galactolipid synthesis genes from photosynthetic gene coexpression lists that have been constructed from the large quantity of *Arabidopsis* transcriptomic data collected to date (ATTED-II, http://atted.jp/).

### TRANSCRIPTIONAL FACTORS INVOLVED IN THE REGULATION OF THE GALACTOLIPID SYNTHESIS GENES AND CHLOROPHYLL SYNTHESIS GENES

We have shown that light signaling via HY5 and cytokinin signaling via AHK2 and AHK3 are involved in upregulation of *MGD1* and *DGD1* during photomorphogenesis (**Figure 3A**). A similar result was observed for *CHLH* expression, whereas only cytokinin signaling had any significant effect on *LHCB6* expression. Genome-wide analysis has identified *DGD1* and *CHLH* as direct targets of HY5 (Lee et al., 2007), and therefore, HY5 may directly upregulate these genes in response to illumination. Because cytokinin is reported to have a stabilizing effect on the HY5 protein (Vandenbussche et al., 2007), it is possible that reduced cytokinin signaling in the* ahk2 ahk3* double mutant led to a decrease in HY5 activity and, thereby, inhibited the upregulation of *DGD1* and *CHLH*. This assumption is consistent with the absence of a BA-induced increase in *DGD1* expression in the *hy5* mutant (**Figure 3B**), suggesting a pivotal role for HY5 in *DGD1* expression during photomorphogenesis. However, BA-induced upregulation of *CHLH* was not repressed in the *hy5* mutant. Moreover, *LHCB6* expression, which is only mildly affected by HY5, also decreased in the *ahk2 ahk3* mutant and increased after BA treatment. These results show that cytokinin signaling can act on photosynthetic gene expression, independently of HY5. In the case of *MGD1*, reduced *MGD1* expression in the *ahk2 ahk3* mutant during photomorphogenesis agrees with similar results in cucumbers, in which cytokinins are essential for the upregulation of cucumber *MGD1* during photomorphogenesis (Yamaryo et al., 2003). In addition, the upregulation of *MGD1* in response to light was attenuated in the *hy5* mutant, even though *MGD1* was not identified as a HY5 target (Lee et al., 2007). Because the HY5 protein is subject to degradation via an E3-ubiquitin ligase CONSTITUTIVE PHOTOMORPHOGENIC1 (COP1) in the dark (Osterlund et al., 2000), it is possible that COP1 downregulates *MGD1* along with other HY5-regulated genes in the dark through degradation of HY5. HY5 is also required for *MGD1* expression in illuminated roots (**Figure 5A**). Considering that COP1 functions in suppressing chloroplast development in roots of light-grown *Arabidopsis* (Deng and Quail, 1992), the COP1–HY5 signaling may also be involved in the regulation of *MGD1* in roots. There has been no in-depth analysis of the *MGD1* promoter region, and therefore we cannot exclude the possibility of direct regulation of *MGD1* by HY5. Alternatively, it is possible that a transcription factor(s) involved in *MGD1* expression requires HY5 to function during photomorphogenesis.

In the 7-day-old seedlings, the expression of *MGD1* and *DGD1* decreased in the *glk1 glk2* double mutant to a greater extent than in the *gnc cga1* double mutant (**Figure 4**), suggesting a stronger impact of the GLK factors on the expression of these genes, as observed in the expression of *LHCB6* and *CHLH*, which are proposed to be direct targets of the GLK factors (Waters et al., 2009). Unlike *LHCB6* and *CHLH*, *MGD1* expression decreased only slightly in the *glk1 glk2* mutant and did not increase in the *GLK* overexpressors, consistent with *MGD1* not being the target of the GLK factors (Waters et al., 2009). Because key genes involved in tetrapyrrole synthesis, including *HEMA1* and *CHLH*, are strongly downregulated in *glk1 glk2,* together with a reduction in chlorophyll contents (Fitter et al., 2002; Waters et al., 2009), it is possible that impairment of tetrapyrrole synthesis would cause the decrease in *MGD1* expression in *glk1 glk2,* as in the *hema1* and *chlh* mutants (**Figure 1B**). Meanwhile, the expression profiles of *DGD1* in *glk1 glk2* and in the overexpressors, were very similar to those of *LHCB6* and *CHLH,* even though *DGD1* has not been identified as a GLK target (Waters et al., 2009). *DGD1* expression also increased in the *GLK1ox* root (**Figure 5B**). These data suggest that GLK factors act as pivotal regulators of *DGD1*, although the mode of action of GLKs in the regulation of *DGD1* remains to be elucidated.

### DISTINCT REGULATION OF GALACTOLIPID SYNTHESIS AND CHLOROPHYLL SYNTHESIS DURING CHLOROPLAST BIOGENESIS IN ROOTS

We reported that chlorophyll synthesis in roots is regulated by auxin/cytokinin signaling at the transcriptional level via the combined action of HY5 and the GLK factors (Kobayashi et al., 2012a). Although the expression of *MGD1* and *DGD1* changed in tandem with *LHCB6* and* CHLH* during leaf development (**Figures 2–4**), *MGD1* and *DGD1* expression in the root differed substantially from *CHLH* expression (**Figure 5A**), showing the uncoupled transcriptional regulation of chlorophyll and galactolipid synthesis during chloroplast biogenesis in the root. Because galactolipids are constitutively required for plastid envelopes, even in non-photosynthetic roots, whereas the requirements of chlorophylls in roots are limited and changeable in response to the growth environment, the transcriptional regulatory systems may differ between these two metabolic processes in non-photosynthetic organs.

In a previous report, we have shown that galactolipids accumulate in the root of *slr-1* mutants without upregulation of *MGD2*, *MGD3*, *DGD1*, and *DGD2* under nutrient-sufficient conditions (Narise et al., 2010). Furthermore, *MGD1* expression in the *slr-1* root remained at the same level as in the wild-type plants (**Figure 5A**), indicating that the increase in galactolipids in the *slr-1* roots was not due to the transcriptional activation of the galactolipid synthesis genes. We did not see a marked upregulation of *MGD1* in the *GLK1ox* root, despite the elevated accumulation of MGDG (**Figure 5B**). It has been reported that plant MGDG synthases are subject to two types of post-translational regulation: the activation by thioredoxins in a redox-dependent manner (Yamaryo et al., 2006; Shimojima et al., 2013) and the activation by anionic phospholipids, phosphatidic acid, and phosphatidylglycerol (Dubots et al., 2010; Shimojima et al., 2013). Therefore, the post-translational activation of MGDG synthesis may contribute to the elevated accumulation of MGDG in the greenish roots. Because it is proposed that the enzymatic regulation of MGDG synthesis activity is not indispensable but is important for the proper development of thylakoid membranes in *Arabidopsis* (Masuda et al., 2011), the post-translational regulation of MGDG synthesis could play a role in coordinating galactolipid synthesis with the formation of photosynthetic machineries, in combination with transcriptional regulation. Several distinct expression patterns between *MGD1* and *DGD1* that were observed in the responsiveness to chloroplast dysfunctions and to the changes in the transcriptional regulators may reflect differences in the involvement of post-transcriptional regulation. This may be the case because *DGD1* expression was more flexible in response to changes in chloroplast development, compared with *MGD1* expression. It is likely that DGDG synthesis by DGD1 is more strongly regulated at the transcription level than is MGDG synthesis by MGD1.

## Conflict of Interest Statement

The authors declare that the research was conducted in the absence of any commercial or financial relationships that could be construed as a potential conflict of interest.
